# Lessons learned from the COVID-19 pandemic on the global health needs of young children: A cross-sectional study

**DOI:** 10.23938/ASSN.1097

**Published:** 2025-01-31

**Authors:** Itsaso Elizalde, Olga Lopez-Dicastillo, Hazel Helen Andueza-Wood, Sara Sola-Cía, Cristina Lozano-Ochoa, Agurtzane Mujika, Naia Hernantes, Beatriz Pereda-Goikoetxea, Elena Antoñanzas-Baztán, María Jesús Pumar-Méndez

**Affiliations:** 1 Public University of Navarra - UPNA Department of Health Sciences Pamplona Navarra Spain; 2 CreaP Research Group- Creación de Capacidad para la Promoción de la Salud Pamplona Navarra Spain; 3 Navarra Institute for Health Research - IdiSNA Pamplona Navarra Spain; 4 La Rioja Center of Biomedical Research - CIBIR Logroño La Rioja Spain; 5 University of the Basque Country - UPV/EHU Faculty of Medicine and Nursing Department of Nursing II Donostia Guipuzcoa Spain; 6 SILO Research Group Spain; 7 Planning, Health Strategy and Research Service Department of Health Government of Navarra Pamplona Navarra Spain

**Keywords:** Preschool Child, COVID-19, Health Promotion, Surveys and Questionnaires, Needs Assessment, Niño en edad prescolar, COVID-19, Promoción de la salud, Encuestas y cuestionarios, Evaluación de necesidades

## Abstract

**Background:**

This cross-sectional study assessed the global health needs of children aged 2 to 6 years and examined how socio- demographic characteristics influenced children’s health needs observed following the COVID-19 pandemic.

**Methodology:**

Cross-sectional study conducted between January and March 2021 in three regions of northern Spain with similar household incomes. Participants were selected through one-stage cluster sampling. A self-reported questionnaire, *Necesidades de salud de la Población Infantil*, was used to assess children’s health needs across seven dimensions and 125 items.

**Results:**

A total of 301 parents or caregivers completed the questionnaire. The dimensions *parental self-efficacy*, *professional advice*, *child socio-emotional aspects*, and *situational influence* were the most frequently reported as significant, while *parent socio- emotional aspects*, *lifestyle*, *promotion of healthy lifestyles*, and *influence of significant people* were the least emphasized. *Life*-*style*-related needs were particularly affected by the limited access to healthcare professionals and were more pronounced when children had a disability.

**Conclusions:**

The findings of this study provide valuable insights for the development of strategies, programs, and interventions aimed at promoting children’s health by addressing the identified needs.

## RESUMEN

**Fundamento:**

Este estudio transversal tiene como objetivo evaluar las necesidades globales de salud de la población infantil de 2 a 6 años y determinar si las características sociodemográficas producen diferencias en las necesidades de salud de los niños observadas después de la pandemia de COVID-19.

**Métodos:**

Estudio transversal realizado en tres regiones del norte de España con ingresos similares. Los participantes se seleccionaron mediante muestreo *cluster* de un paso. El cuestionario auto respondido por los padres *Necesidades de salud de la Población Infantil* evaluó las necesidades de salud mediante siete dimensiones y 125 ítems.

**Resultados:**

Un total de 301 progenitores/cuidadores completaron el cuestionario. Las dimensiones *autoeficacia parental, asesoramiento profesional, aspectos socioemocionales del niño* e *influencia situacional* alcanzaron las puntuaciones más altas, mientras que *aspectos socioemocionales de los padres, estilo de vida, promoción de estilos de vida saludables* e *influencia de personas significativas* recibieron las puntuaciones más bajas (mayores necesidades). Las necesidades relacionadas con el estilo de vida se vieron afectadas por la falta de acceso a profesionales de la salud y con mayor intensidad cuando los niños tenían una discapacidad.

**Conclusiones:**

El resultado de este estudio podría enriquecer la planificación de estrategias, programas e intervenciones para promover la salud infantil, cubriendo las necesidades identificadas.

## INTRODUCTION

Children’s health is defined as the extent to which they are able to develop and achieve their potential, fulfill their needs, and build the capacities that enable them to interact successfully with their environment^1^. Caring for children’s health is essential for enhancing their immediate and long-term development, contributing to societal prosperity and the sustainability of health systems^2^.

During the COVID-19 pandemic, children faced significant disruptions in healthcare access, pediatric services were scaled back to accommodate the surge in adult COVID-19 hospital admissions. Routine face-to-face appointments and check-ups were either cancelled or moved to online consultations. More importantly, schools, nurseries, and early childhood education centers were close and social distancing measures were enforced^3^. These disruptions had a substantial impact on children’s health, with an increase in the use of electronic devices and changes in sleep and eating patterns. Fear and anxiety also escalated during the lockdown, negatively affecting both children’s and families’ well-being. The impact of these measures was influenced by factors such as age, educational level, pre-existing mental health conditions, economic status, direct COVID-19 infection, and the fear of being infected^4^. These factors significantly affected children’s health, with the greatest consequences for those living in disadvantaged conditions^5^^,^^6^^,^^7^.

In response to this situation, evidence suggests that health promotion could be an effective strategy for improving children’s health and addressing the underlying axes of inequality^8^. The success of health promotion strategies and interventions depends not only on incorporating a balanced mix of these actions but also on ensuring their design is grounded in specific health promotion models that address the health needs of the target population^9^.

Health needs can be defined as *situations or characteristics that are desirable to modify and transform towards outcomes and benefits in terms of health and quality of life*^10^. Children’s dependence on families and communities to meet these needs makes them particularly vulnerable and susceptible to environmental influences^11^, such as those experienced during the COVID-19 pandemic. Additionally, pediatric developmental processes involve a complex interplay of biological, behavioral, social, and environmental factors^12^, which results in a wide range of health needs depending on the child’s stage of development.

Published studies on the effects of the COVID-19 pandemic on children’s health needs have typically included children treated as general population, often including both young and older age groups together or focusing only on older children. As a result, the distinct health needs of younger children have been underrepresented^13^^,^^14^. Furthermore, most publications address specific aspects of children’s health needs rather than assessing them comprehensibly. For instance, studies have focused on mental health issues among children and parents^13^^,^^15^^,^^16^, their well-being^17^, or lifestyle factors such as diet or physical activity, often in relation to conditions like unhealthy weight^18^^,^^19^.

Comprehensive assessments on global health needs in young children is challenging. However, a useful tool for this purpose is the *Necesidades de salud de la Población Infantil* (Health needs in child population, *NPI*) questionnaire^20^. This online, parent self-report tool, validated for children aged 2 to 6 years, is mainly useful for assessing global health needs. The *NPI* questionnaire proved especially valuable during the post-pandemic period when face-to-face consultations became more difficult, enabling the analysis of various needs within the population^21^. Furthermore, its accessibility -allowing parents to complete it on any device- made it made more available to families, despite the survey’s length.

This study was designed to fill gaps in the literature by assessing the global health needs of young children from diverse backgrounds after the COVID-19 pandemic, providing a foundation for the development of future health promotion strategies. The primary objective was to assess the health needs of children aged 2 to 6 years, explore potential differences based on sociodemographic factors, and examine how limited access to healthcare and education professionals affects these health needs.

## METHODS

### Design and setting

This cross-sectional study was conducted between January and March 2021 in three regions of northern Spain that share similar socioeconomic characteristics and are geographically close (1 - Basque Country: € 37,598; 2 - Navarre: € 37,728; 3 - La Rioja: € 32,096). These regional income levels are higher than the Spanish national average (€ 30,552)^22^.

### Participants and sampling

The study was introduced to the Education Department of each regional government, requesting their collaboration to facilitate access to randomly selected parents of children aged 2 to 6 years through schools, nurseries, and early childhood education centers. Following this, the principals of the centers were contacted via email and phone to explain the study and the level of participation required from the parents who agreed to take part.

A one-stage cluster sampling method was used to select participants. The sample size was calculated using the sample-size.net calculator (https://sample-size.net/means-effect-sizeclustered/), with a significance level of α = 0.05, an average of 14 children per cluster (with a response rate of 35%), and an intraclass correlation coefficient (rho) of 0.2. Based on these parameters, it was estimated that 300 surveys across 60 clusters would be required to detect moderate differences between groups in dimensions derived from the *NPI* questionnaire. This calculation was based on a Cohen’s d effect size of 0.5, assuming an unequal distribution of participants between groups (25% vs. 75%). The sample size was also sufficient to identify smaller differences (Cohen’s d effect size of 0.435) when the groups were evenly distributed (50% vs. 50%).

From the list of all groups obtained from each regional government archive, 42 groups in each of the three regions were randomly selected using SPSS. Some of the 126 selected groups refused to participate after the initial contact; thus, a second round of random sampling without replenishment was performed following the same procedure as in the first round.

### Data collection

The centers distributed information about the study and a link to the questionnaire (through Surveymonkey^®^) to the families of the selected groups. Each center determined the method of distribution (email, online platforms, etc.). Contact details of the research team were provided in case any family wished to seek further information or assistance in completing the questionnaire. Parents who agreed to participate accessed the link and completed the questionnaire anonymously, without providing any identifying information about themselves or their children.

### Instrument

Data were collected using the parent-reported *Necesidades de salud de la Población Infantil* (*NPI*) questionnaire^20^. This instrument demonstrates excellent internal consistency (Cronbach’s α = 0.979) and good model fit for a 7-factor structure (RMSEA = 0.048), with normed fit index (NFI) = 0.741 and comparative fit index (CFI) = 0.779. The questionnaire includes 119 items, 23 of which are nominal-scale questions to gather sociodemographic data. For this study, six additional questions were included to capture information on changes in the employment status of family members due to the pandemic and the accessibility of health and educational centers during that time.

The seven dimensions related to factors influencing children’s health needs consist of 96 items, which are assessed using Likert-type scales with five response options. Higher scores indicate lower health needs:

- *lifestyle* (21 items, score: 21-105; α = 0.964): evaluates both children’s and parents’ habits related to nutrition and exercise, with representative items addressing the frequency of physical activity and dietary choices, such as fruit and vegetable intake;
- *promotion of a healthy lifestyle and influence of significant people* (22 items, score: 22-110; α = 0.962): explores the impact of influential figures, such as parents or friends, on children’s health behaviors, asking questions about how parental habits or advice from peers shape choices around exercise and eating;
- *children’s socioemotional aspects* (18 items, score range: 18-90; α = 0.900): assesses emotional and social behaviors in the children, such as their ability to express feelings and interact with peers, with items addressing mood swings or responses to social situations;
- *parents’ socioemotional aspects* (5 items, score range: 5-25; α = 0.940): assesses parental emotional well-being and its potential impact on parenting practices, including questions about stress levels and perceived support;
- *parental self-efficacy* (15 items, score range: 15-75; α = 0.993): focuses on parents’ confidence in managing their children’s health and behaviors, with items examining their ability to encourage healthy eating or manage behavioral challenges;
- *situational influences* (10 items, score range: 10-50; α = 0.969): examines external factors that affect health behaviors, such as access to healthcare or community resources, including questions about barriers like the availability of parks or health services;
- *professional advice* (5 items, score range: 5-25; α = 0.973): evaluates the guidance provided by healthcare professionals and educators, with items assessing the frequency and usefulness of advice from doctors, teachers, or nurses;

Together, these dimensions provide a comprehensive assessment of children’s health, with a total score range of 96-480.

### Variables

The following variables were collected:

- Demographic variables: relationship to the child (mother, father, other); place of origin (Spain, other); age group (<38 years, 38-41 years, >41 years); level of education (primary, secondary, vocational training, university, master/PhD); Region (1: Basque Country, 2: Navarre, 3: La Rioja); population size in area of residence (urban: >10,000 inhabitants, suburban: 2,000-10,000 inhabitants, rural: <2,000 inhabitants), household size (2, 3, 4, 5, 6 members); time spent with the children (weekends only, up to 2 hours per day, >2 hours per day); sex of child (boy, girl).
- Health during childhood: chronic illness (yes, no); disability (yes, no); body mass index (BMI) (normal weight, underweight, or overweight/malnutrition).
- Social and economic factors: respondent’s and partner’s employment status at the time of the questionnaire (employed, unemployed); number of hours worked per week (<20 hours, 20-40, >40hours); change in respondent’s and partner’s employment status during the pandemic (no change, temporary layoff, unemployed, found work); level of family income per month (<1,000 €, 1,000-5,000 €, >5,000 €); changes to employment situation due to pandemic measures (temporary layoff, lost job, other [please specify], working hours increased, working hours reduced).

### Ethical aspects of the research

This study was reviewed and approved by the local ethical research committee (*Comité de Ética de la Investigación con medicamentos de Navarra*; PI_2020/105). Written information about the study was provided to participants along with the link to the questionnaire, and their consent to participate was implied upon submission of their responses. Participants were informed about the confidentiality of their responses and were assured that all data would be anonymized in accordance with the Spanish personal data protection law^23^.

### Data analysis

Quantitative variables were presented as arithmetic means and standard deviations (SD) if they followed a normal distribution, and as medians and interquartile ranges (IQR) if they did not; normality was assessed using the Shapiro-Wilk test. Categorical variables were described as frequency and distribution. As quantitative variables were not normally distributed, non-parametric tests were used to compare variables between two independent groups (Mann-Whitney U) or more independent groups (Kruskal-Wallis). P-values less than 0.050 were considered statistically significant. The data were analyzed using STATA/SE v16.1.

## RESULTS

Initially, 126 groups were selected, of which 24 declined to participate: 18 from Region 1 (75%), four from Region 2 (16.7%), and two (8.4%) from Region 3. The reasons for not participating were: 1) the language of the *NPI* questionnaire (Spanish) (n = 9; 37.5%), 2) participation in other similar studies and/or survey saturation during the pandemic (n = 5; 20.9%), and 3) not having children within the target age group at the time of the study (n = 2; 8.3%). Eight groups (33.3%) did not provide a specific reason for declining.

In the second round of sampling, 11 centers (78.6%) were from Region 1, two from Region 2, and one from Region 3). The questionnaire was ultimately sent to 116 groups comprising 1,940 families with children aged between 2 to 6 years. Access to data from non-participating individuals was not possible.

A total of 352 families returned the questionnaire, resulting in a response rate of 18.14%. Of these, 301 questionnaires (85.5%) were fully completed, completed with the majority by the mother (n = 265; 88.04%) and three (1%) by other caregivers or guardians. The distribution by region was as follows: Region 1 (n = 109; 36.45%), Region 2 (n = 79; 26.43%), and Region 3 (n = 111; 37.12%).

Most respondents were mothers (88.04%), of Spanish origin (90.70%), living in urban areas (65.32%), under 38 years of age (40.86%), and had university-level studies (46.18%). The most common household sizes were four members (60.87%) or three members (24.41%). In general, adults reported spending more than two hours per day with their child (76.41%). A significant percentage of children were reported to be either underweight or overweight (40.53%). Additionally, 4.65% of the children had a chronic illness, and 2.33% had a disability.

At the time the questionnaire was completed, the employment status of over 90% of the respondents and their partner remained similar to that at the beginning of the pandemic. A total of 85.38% of the respondents were employed, with 78.91% working 20-40 hours per week. Most partners (93.66%) were also employed, with 31.73% working over 40 hours per week. Approximately 90.22% of respondents reported a monthly family income between 1,000 and 5,000 euros (Table 1).

**Table 1:** Characteristics of the respondents and children [n (%)]

Demographic		Economic	
*Relationship with the child*		*Level of family income per month*	
Mother	265 (88.04)	<1,000 €	11 (3.99)
Father	33 (10.96)	1,000-5,000 €	249 (90.22)
Other	1 (1.00)	>5,000 €	16 (5.8)
*Origin*		Employment	
Spain	273 (90.7)	*Respondent’s status when the questionnaire was completed*	
Other	28 (9.3)	Employed	257 (85.38)
*Age*		*Hours worked per week*	
<38 years	123 (40.86)	<20 hours	19 (7.42)
38-41 years	91 (30.23)	20-40 hours	202 (78.91)
>41 years	87 (28.91)	>40 hours	35 (13.67)
*Level of education*		*Changes to employment situation during pandemic*	
Primary	6 (1.99)	No change	275 (91.97)
Secondary	22 (7.31)	Temporary layoff	12 (4.01)
Vocational training	98 (32.56)	Unemployed	11 (3.68)
University	139 (46.18)	Found work	1 (0.33)
Masters/PhD	36 (11.96)	Partner’s Status when the questionnaire was completed	
*Area of residence*		Employed	266 (93.66)
Urban	194 (65.32)	Hours worked per week	
		<20 hours	14 (5.17)
Suburban	62 (20.88)	20-40 hours	171 (63.1)
Rural	41 (13.8)	>40 hours	86 (31.73)
*Household size*		*Changes to employment situation during the pandemic*	
2	8 (2.68)	No change	155 (92.26)
3	73 (24.41)	Temporary layoff	7 (4.17)
4	182 (60.87)	Unemployed	6 (3.57)
5	27 (9.03)	Family	
6	9 (3.01)	*Changes to employment situation during the pandemic*	
*Time spent with children*		Temporary layoff	13 (5.75)
Weekends only	14 (4.65)	Lost their job	11 (4.87)
Up to 2 h/day	57 (18.94)	Other	117 (51.77)
>2 h/day	230 (76.41)	Working hours increased	55 (24.34)
Child		Working hours reduced	30 (13.27)
Sex (girl)	230 (76.41)		
Chronic illness	14 (4.65)		
Disability	7 (2.33)		
*Body Mass Index*			
Normal weight	179 (59.47)		
Under/overweight	122 (40.53)		

**Figure 1:**
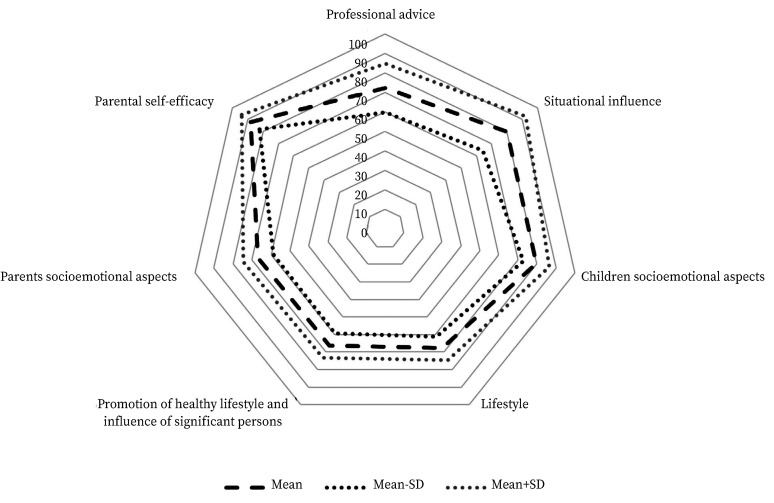
Overall scores and standard deviation for the seven dimensions of questionnaire.

The areas of the *NPI* questionnaire where respondents scored more positively included parental self- efficacy, children’s socioemotional aspect*s*, professional advice, and situational influence. Conversely, the lowest scores were observed in the dimensions of parent’s socioemotional aspects, lifestyle promotion, and health lifestyle promotion, and the influence of significant others. In several areas, a high standard deviation from the mean was noted, indicating that the data were widely dispersed (Fig. 1).

Table 2 presents the scores for the seven dimensions of the questionnaire, as well as the overall scores, categorized by sociodemographic characteristics. Differences based on the respondent’s age were observed in the dimensions of *promotion of healthy lifestyle and influence of significant others*, with older parents scoring significantly lower than younger parents (median = 70; IQR: 66-76 vs 73; IQR: 69.5-79; p = 0.038).

Significant regional differences were seen for some dimensions. Specifically, Region 3 scored lower than Region 1 in *parental self-efficacy* (66; IQR: 62-68 vs 67.5; IQR: 64-70; p = 0.017), while Region 1 had the lowest scores for *situational influences* (21.5; IQR: 19-24; p = 0.004).

In contrast, higher scores were obtained for the *professional advice* dimension for participants from Region 3 (38; IQR: 34-41) compared to those from Regions 1 and 2, whose scores ranged from 35 to 37 (p = 0.143). Additionally, scores for *professional advice* differed depending on participants’ educational level, with those holding a university degree, master’s degree, or doctorate scoring lower than those with only primary or secondary education (35; IQR: 31-40 vs 38.5; IQR: 35-43; p = 0.006).

Parents who spent more than two hours per day with their child exhibited significantly higher median scores in two dimensions compared to those who only spent weekends with their child: *children’s socioemotional aspects* (73; IQR: 68-77 vs 68.5; IQR: 65-73; p = 0.027) and *parental self-efficacy* (67; IQR: 64-70 vs 65.5; IQR: 63-70; p = 0.009). Participants with a child with a disability scored lower on the dimension *promoting a healthy lifestyle* (64; IQR: 61-69 vs 73; IQR: 68.78; p = 0.032). No significant differences were observed in the median scores for any dimension based on participants’ origin, the size of the population in which the families lived, the presence of a chronic condition, or participants’ BMI.

**Table 2:** Impact of sociodemographic characteristics on the dimensions of the questionnaire

	Scoring for the dimensions / Median (IQR)							
Characteristics	Professional advice	Children’s socioemotional aspects	Parental self-efficacy	Lifestyle	Situational influences	Promotional healthy lifestyle	Parent’s socioemotional aspects	Total
*Origin*	p = 0.765	p = 0.078	p = 0.285	p = 0.633	p = 0.841	p = 0.574	p = 0.105	p = 0.636
Spain	37 (33-40)	72 (68-76)	66 (63-69)	71.5 (67-76)	21 (18-24)	73 (67-79)	17 (15-18)	354 (343-369)
Other	36 (32-40)	73 (72-79)	67.5 (66-70)	73 (68-75)	20 (18-22)	72 (70-73)	16 (14-18)	354 (346-365)
*Age (years)*	p = 0.766	p = 0.902	p = 0.414	p = 0.440	p = 0.314	p = 0.038	p = 0.799	p = 0.610
<38	36 (32-40)	72 (68-76)	66 (64-70)	71 (66.5-75)	21 (18-23)	73 (69.5-79)	16 (15-18)	355 (344-369)
38-41	36 (32.5-41)	72 (68-76)	66 (64-69)	72 (67-75)	21 (17-24)	72 (67.5-79)	16.5 (15-18)	354 (344-369)
>41	37 (33-40)	73 (67-77)	66 (63-69)	73 (67-76.5)	20 (16-22)	70 (66-76)	17 (16-18)	354 (341-367)
*Education level*	p = 0.006	p = 0.501	p = 0.841	p = 0.587	p = 0.991	p = 0.125	p = 0.318	p = 0.618
Primary/secondary	38.5 (35-43)	73 (69.5-79.5)	67 (63-69)	72 (67-76)	20 (17-24)	71 (67-73)	16 (15-17)	356.5 (344-371)
Vocational training	38 (34-40)	73 (68-76)	66 (63-70)	73 (66.5-76.5)	21 (18-23)	72 (66-77)	17 (15-18)	356 (346-367)
University	35 (31-40)	72 (68-76)	67 (64-69)	71 (67-75)	21 (18-23)	73 (69-79)	16 (15-18)	353 (342-370)
*Region*	p = 0.143	p = 0.121	p = 0.017	p = 0.109	p = 0.004	p = 0.849	p = 0.694	p = 0.268
1	37 (31-40)	73 (69-77)	67.5 (64-70)	71.5 (67-76)	21.5 (19-24)	73 (67-78)	17 (15-18)	356 (345-371)
2	35 (32-39)	72 (66-76)	66 (63-69)	73 (68-76.5)	21 (18-23)	71 (68-78)	16 (16-18)	353 (344-364)
3	38 (34-41)	72 (67-76)	66 (62-68)	71 (66-75)	20 (16-22)	72.5 (67.5-79)	16 (15-18)	353 (341-365)
*Area of residence*	p = 0.416	p = 0.844	p = 0.233	p = 0.307	p = 0.803	p = 0.941	p = 0.477	p = 0.988
Urban	36 (32-40)	73 (68-76)	67 (64-69)	72 (67-76)	21 (18-24)	73 (68-78)	16 (15-18)	354 (344-369)
Semirural	38 (33-41)	72 (68-76)	65 (63-69)	71 (63-75)	20 (18-23)	72 (67-79)	17 (15.5-18)	354.5 (340-367)
Rural	36 (34-40)	72 (68-78)	65.5 (63-69.5)	73 (68-76)	20 (17.5-22)	72 (68-79)	16 (15-18)	352.5 (345-368)
*Time spent with the child*	p = 0.151	p = 0.027	p = 0.009	p = 0.052	p = 0.411	p = 0.384	p = 0.113	p = 0.051
Weekends only	40 (35-45)	68.5 (65-73)	65.5 (63-70)	66 (63-68)	20 (16-21)	71 (67-76)	18 (16-20)	344 (330-370)
Up to 2 hours per day	35 (32-40)	71 (67-75)	65.5 (60-68)	70 (67-75)	20 (17-22)	71 (69-76)	16.5 (16-18)	348 (344-357)
>2 hours per day	37 (33-40)	73 (68-77)	67 (64-70)	72 (67-76)	21 (18-24)	73 (67-79)	16 (15-18)	356 (344-370)
*Chronic illness*	p = 0.503	p = 0.492	p = 0.322	p = 0.012	p = 0.053	p = 0.690	p = 0.603	p = 0.102
No	37 (33-40)	72 (68-76)	66 (64-69)	72 (67-76)	21 (18-23)	72 (68-78)	16 (15-18)	354 (344-369)
Yes	34 (33-38.5)	71 (69-73)	66 (58-68)	69 (63-72)	18 (16-21)	72 (61-80)	16.5 (16-17)	342 (329-353)
*Disability*	p = 0.819	p = 0.221	p = 0.103	p = 0.769	p = 0.926	p = 0.032	p = 0.169	p = 0.645
No	37 (33-40)	72 (68-76)	66 (64-69)	72 (67-76)	21 (18-23)	73 (68-78)	17 (15-18)	354 (344-369)
Yes	34 (33-40)	65 (53-78)	61 (58-67)	70 (69-72)	20.5 (19-22)	64 (61-69)	15 (14-17)	352 (326-370)
*Body Mass Index*	p = 0.324	p = 0.431	p = 0.366	p = 0.483	p = 0.078	p = 0.721	p = 0.478	p = 0.319
Normal weight	37 (33-41)	72 (68-76)	66 (64-70)	71.5 (67-76)	21 (18-24)	73 (69-78)	16 (15-18)	355 (344-370)
Under/overweight	36 (32-39)	73 (68-77)	66 (63-69)	72 (68-76)	20 (18-22)	72 (67-79)	17 (15-18)	353 (343-367)

IQR: interquartile range.

Although changes in parents’ employment status were observed, with 4.87% of respondents reporting job loss during the pandemic and 5.75% being temporarily laid off, no significant differences were found in any of the dimensions of children’s health needs (Table 3). Regarding participants’ employment status and family income levels, differences were found only in the parental socioemotional dimension, where families with the lowest or highest incomes levels scored lower (p = 0.041), as shown in Table 3.

**Table 3:** Impact of economic and employment characteristics on the dimensions of the questionnaire

	Scoring for the dimensions / Median (IQR)							
Employment characteristics	Professional advice	Children’s socioemotional aspects	Parental self-efficacy	Lifestyle	Situational influences	Promotional healthy lifestyle	Parent’s socioemotional aspects	Total
Employed*	p = 0.624	p = 0.426	p = 0.566	p = 0.115	p = 0.089	p = 0.294	p = 0.980	p = 0.311
Yes	37 (32-40)	72 (68-76)	66 (64-69)	71 (67-76)	21 (18-24)	73 (68-78.5)	17 (15-18)	354 (343-369)
No	36 (34-40)	73 (70-77)	66 (62-69)	73 (69-76)	19 (17-23)	71 (67-78)	16 (15-18)	356 (249-368)
*Employment status during the pandemic*	p = 0.080	p = 0.188	p = 0.205	p = 0.136	p = 0.679	p = 0.673	p = 0.404	p = 0.235
No change	36 (33-40)	72 (68-76)	66 (63-69)	72 (67-76)	21 (18-23)	72.5 (68-78)	17 (15-18)	354 (343-369)
Temporary layoff	38.5 (38-40)	71 (64-76)	69 (68-70)	69 (65-75)	19 (17-23)	76 (72-81)	16 (15-16)	349 (346-356)
Unemployed	40 (36-46)	76 (72-79)	67 (66-70)	75.5 (72-84)	20 (19-22)	72 (67-80)	16 (16-18)	365 (352-391)
*Level of family income*	p = 0.592	p = 0.197	p = 0.555	p = 0.505	p = 0.802	p = 0.194	p = 0.041	p = 0.072
<1,000 €	37 (35-43.5)	76 (72-80)	67 (63-70)	68 (66-75)	21 (16.5-23-5)	73 (20.5-80.5)	16 (15-18)	359 (353-369)
1,000-5,000 €	36 (33-40)	72 (68-76)	66 (63-69)	72 (68-76)	21 (18-23)	73 (68-79)	17 (15-18)	355 (344-370)
>5,000 €	35.5 (32-40)	70.5 (66-76)	65 (64-68)	69 (65-75)	21.5 (18-24)	69.5 (67-72)	16 (14-16)	347 (334-356)

IQR: interquartile range; *: at the time of questionnaire.

Finally, the relationship between limited access to health and education professionals and children’s health needs was explored by analyzing the association between the questionnaires’ dimensions and the two items presented in Table 4. No significant relationships were found for any dimension except for the accessibility of health professionals, which was significantly related to the lifestyle dimension (p = 0.029).

**Table 4:** Score [median (IQR)] in additional questions regarding the exceptional situation caused by the COVID-19 pandemic

	Completely disagree	Disagree	Not sure	Agree	Completely agree	p- value
*The pandemic has affected the accessibility to my child’s education professionals*						
Professional advice	39.5 (34-42)	36.5 (34-40)	33 (31-40)	36 (33-40)	36.5 (31-40)	0.155
Children’s socioemotional aspects	72 (67-76)	72.5 (68-76)	73 (70-74)	73 (68-76)	74 (6978)	0.738
Parental self-efficacy	68 (65-70)	66 (64-70)	66 (64-68	66 (62.5-69)	67 (65-70)	0.214
Lifestyle	70 (66-76)	71.5 (67-76)	74 (69-75)	72 (68-75)	71 (67-75)	0.769
Situational influences	22 (19-24)	20 (18-23)	20 (18-22)	20 (17-23)	19 (16-23)	0.212
Promotional healthy lifestyle	75 (66-78)	71 (66-78)	73 (68-82)	72 (68-79)	73 (70-78)	0.675
Parent’s socioemotional aspects	17 (15-18)	16 (15-18)	17 (16-18)	16 (15-18)	17 (16-18)	0.243
Total	356.5 (342.5-374)	354 (340-364)	352 (343-362)	353 (346-370)	357 (344.5-367)	0.719
*The pandemic has affected the accessibility to my child’s health professionals*						
Professional advice	NA	44 (29.5-46)	35 (32-38)	38 (34-40)	37 (33-40)	0.150
Children’s socioemotional aspects	72 (69-77)	76.5 (72-79)	72 (68-75)	73 (67-73)	71 (66-76)	0.238
Parental self-efficacy	65 (61-68)	67.5 (67-71)	66 (63-69)	66 (64-69)	66 (62-69)	0.093
Lifestyle	74 (67-76)	77.5 (71-81)	71 (68-75)	73 (68-76)	73 (68.5-76)	0.029
Situational influences	21 (19-24)	22 (21-24)	20.5 (18-24)	20 (19-22)	20 (17-23)	0.750
Promotional healthy lifestyle	67 (67-67)	72 (62-79)	70.5 (65.5-77)	72.5 (68-74.5)	72 (68-76)	0.278
Parent’s socioemotional aspects	16 (14-18)	16.5 (16-18)	16 (15-17)	17 (16-18)	16 (15-18)	0.712
Total	NA	370 (352-374)	352 (340-364)	354.5 (349-362.5)	353 (343-369)	0.299

IQR: interquartile range; NA: not applicable/unable to calculate due to insufficient data.

## DISCUSSION

By using the *NPI* tool, this study collected data that offer a comprehensive overview of young children’s health needs during the COVID-19 pandemic. Overall, the dimensions with the highest scores, and thus those identified as strengths, are professional advice, the child’s socioemotional development, parental self-efficacy, and situational influences.

The dimension *professional advice* is influenced by parental education level, with university-educated respondents scoring lower. This finding suggests that this group of parents either felt they lacked the adequate advice or identified more unmet needs compared to parents with lower education levels. Notably, previous research has shown that parents with university-level education are more likely to seek professional advice when their child is unwell, demonstrating higher levels of engagement and utilization of child health services^24^. In contrast, individuals with limited literacy skills tend to engage with traditional health education messages, underutilize preventive health services, and face greater challenges in managing chronic illnesses^25^. Furthermore, parents’ responses may have been influenced by difficulties in obtaining timely professional advice due to pandemic-related restrictions, as indicated both this study and the existing literature^26^.

The findings related to the dimensions of children’s socioemotional aspects are notably positive, suggesting that the socioemotional needs of 2- to 6-year-old children were largely met during the pandemic^27^. These results highlight the important role parents play in protecting children from the stress caused by the COVID-19 pandemic. Parent’s socioemotional aspects, such as maintaining home routines, being available to discuss the pandemic with their children, and assisting them in recognizing their emotions, are identified as contributors for effective parental buffering of stress. The opportunity for families to spend more time together during the COVID-19 pandemic has been recognized as a positive factor in several studies^28^^,^^29^. This finding is further supported in this study, where parents who reported spending more time with their children obtained higher scores on the dimension of children’s socioemotional aspects. However, it is important to consider that the positive effects of family time may be moderated by stress levels, particularly in households facing additional challenges. Families experiencing job loss, income reductions, caregiving burden, and illness may not experience the same benefits, as financial stress and other burdens can overshadow the potential advantages in increased family time, leading to substantially worse well-being compared to those without such hardships^29^.

An increase in the amount of time adults spend with their children also positively influences another highly rated dimension: parental self- efficacy. Family time contributes to stronger family relationships, which are crucial for a healthy development of young children- Emotionally significant and supporting relationships have being linked to better physical and psychological health in adolescence and adulthood^30^. Additionally, the region in which participants resided influenced parental self-efficacy. Specifically, individuals from regions with lower economic status reported the lowest scores for parental self-efficacy. This finding is in line with previous research indicating that financial factors, such as household income and socioeconomic status, can affect parental self-efficacy^31^.

This pattern is consistent with the additional finding that economic status also plays a significant role in producing higher situational influence scores. These consistent relationships highlight the critical role of socioeconomic factors in shaping children’s living environments, which, in turn, directly affect their needs and development^32^. Research has shown that children from lower-income families are more likely to experience adverse health outcomes, including lower levels of physical activity and poorer dietary habits^33^. A recent report further revealed that children’s health needs are particularly affected in socioeconomically disadvantaged populations, underscoring the significant influence and socioeconomic disparities on children’s developmental environments^34^. This highlights the urgent need to address these disparities, emphasizing their essential role in promoting equity in child health and development, as well as improving long-term outcomes.

The lowest score observed in this study is for the parental socioemotional dimension. High levels of parental burnout have been related with lockdown and the lack of personal space in the home environment^35^, as well as with remote work while caring for young children^36^. Additionally, most participants in this study were mothers, and previous research has highlighted gender differences, particularly the impact during of the COVID-19 pandemic on women’s mental health and well-being^37^. While the time spent on childcare significantly increased for families during the pandemic, this increment disproportionately affected women, contributing to a re-traditionalization of gender roles^38^. This shift in roles adds greater stress on mothers, especially those lacking support or having to manage home-schooling responsibilities^39^. Although the gender gap in caregiving responsibilities is well documented, with women generally spending more time on child-rearing activities than men do^38^, no significant differences are seen between women and men in this study. This may be attributed to the study’s sample, in which only 10% of respondents were fathers. Future research should address this gender imbalance in parenting representation and strive for more balanced participation to gain a comprehensive understanding of parental experiences.

The increase in parental stress and emotional disorders during the pandemic has been shown to negatively affect parenting capabilities, with evidence suggesting that it can ultimately lead to less responsive and affectionate parenting^40^^,^^41^. These findings underscore the urgent need to prioritize accessible mental health support for parents as they navigate the aftermath of the pandemic, in order to prevent potential long-term repercussions for children.

Once again, family income is identified as an influential factor affecting the parental socioemotional dimension. Numerous studies have demonstrated the high prevalence of financial stress during the pandemic, with socioeconomically vulnerable families being most adversely affected^42^^,^^43^^,^^44^. However, in the present study, parents earning less than €1,000 are impacted to the same extent as those earning more than €5,000. Financial stress has been linked to factors beyond income level, such as a lack of savings or increased debt^45^. Future studies should explore these additional variables to provide further insight into these findings.

Low scores are observed for two other dimensions, specifically lifestyle (promotion of healthy lifestyles) and the influence of significant others, thus identified as weaknesses. Previous research has shown a decline in physical activity levels of young children during the pandemic, along with changes in sleep patterns, among other lifestyle adjustments^46^. Lockdowns and social restrictions, implemented to curb the spread of the SARS-CoV-2 virus, may have made it difficult for parents of young children to maintain regular recreational activities, physical exercise, and proper nutrition^47^. Additionally, families may have faced challenges accessing healthcare professionals or receiving support, particularly regarding their children’s lifestyle-related concerns. This conclusion is supported by the correlation between the pandemic’s impact on parents’ access to healthcare professionals and the low scores in the dimension *lifestyles*. This finding highlights that neglecting health during the pandemic was not inconsequential, underscoring the need for future contingency plans that specifically address the promotion of health during similar crises.

Notably, parents of children with a disability scored particularly low regarding the promotion of healthy lifestyles and influence of significant people. Previous studies have reported that parents of children with disabilities faced a lack of support and feelings of isolation from healthcare professionals during the COVID-19 pandemic^48^. Children with disabilities may have had a greater degree of unmet needs in this regard compared to children without disabilities.

This study has several limitations. Given that the target population consisted of 2- to 6-year-old children, parental feedback was used to collect data, as it is challenging for young children to complete complex questionnaires. Future studies should focus on developing new tools in a developmentally appropriate format to enable children to actively participate in research. Additionally, this study was conducted using online resources due to the impact of COVID-19. The concurrent conduct of many studies during this period may have influenced the response rate. A low response rate may introduce biases, as the characteristics and views of non-respondents may differ from those who participated. This may reduce the representativeness of the sample, potentially skewing the findings and limiting the generalizability of the conclusions to the broader population. For example, families with limited access to the Internet or technology may not have received the invitation to participate or were unable to complete the questionnaire, which could lead to a misrepresentation of this population and contribute to the lower response rate. Face-to-face interactions, as demonstrated in previous studies using the NPI questionnaire^20^, may help increase response rates. Another limitation is the lack of detailed data on non-participants, which makes it difficult to fully understand how participants differ from non-participants. However, careful registration of non-participation reasons suggest that non-participation may not have introduced systematic bias into the study outcomes, as similar groups were included in the sample afterwards.

Despite these limitations, the study successfully achieved its target sample size. More than 300 parents completed the questionnaire, resulting in a response rate of 15.52% for the initially proposed sample. This is a notable accomplishment, especially considering the extensive length of the questionnaire. Although the survey’s length may have discouraged some parents from participating, it enabled a comprehensive understanding of the current global health needs of young children, providing a novel and valuable perspective to inform the development of improved health promotion strategies.

One notable strength of the study lies in its use of a one-stage cluster sampling method. During the pandemic, surveys proliferated through communication platforms like *WhatsApp*, reaching a wide range of the public and employing a mix of convenience and snowball sampling techniques. While these methods have the potential to generate a large volume of responses, they also hinder the accurate determination of the true response rate, introduce selection and response biases, and limit the generalizability of findings due to potential issues with sample representativeness^49^. In contrast, by avoiding these sampling limitations, the present study gains greater rigor, thereby enhancing its overall validity.

This study provides a comprehensive analysis of young children’s health needs during the pandemic. The results reveal that while the socioemotional needs of these children are generally met, their lifestyle needs and promotion are the most significally impacted. Parents are able to address their children’s socioemotional needs, with *parental self-efficacy* being one of their strengths, despite their own socioemotional needs being compromised. Both low- and high-income families face greater needs. Children’s lifestyle needs are particularly affected by a lack of access to healthcare professionals, with children with disabilities being more strongly impacted. The region in which the families lived also influence situational and parental self-efficacy needs. These findings offer valuable insights that may help enrich strategies, programs, and interventions aimed at promoting children’s health by addressing the identified needs.

### Relevance for clinical practice

Nurses and health professionals caring for families with young children can use the findings of this study to tailor their care using a holistic approach. The study shows that, despite their own struggles, parents effectively addressed their children’s socioemotional needs during the pandemic. However, children’s lifestyle needs suffered due to limited access to healthcare professionals, particularly that of children with disabilities. Income disparities and regional factors also play a role. Assessing children’s overall health needs allows for the identification of areas were interventions may unnecessary, as well as those where resources can have greater impact on promoting children’s health, ultimately contributing to the sustainability of healthcare systems.
